# Tracheostomy in intensive care unit patients can be performed without bleeding complications in case of normal thromboelastometry results (EXTEM CT) despite increased PT-INR: a prospective pilot study

**DOI:** 10.1186/s12871-015-0073-1

**Published:** 2015-06-10

**Authors:** Miroslav Durila, Pavel Lukáš, Marta Astraverkhava, Jan Beroušek, Michal Zábrodský, Tomáš Vymazal

**Affiliations:** 1 Department of Anaesthesiology and Intensive Care Medicine, Second Faculty of Medicine, Charles University and University Hospital Motol, V Uvalu 84, 15006 Prague, Czech Republic; 2 Clinic of Otorinolaryngology and Surgery of the Head and Throat of the First Faculty of Medicine of Charles University and University Hospital Motol, V Uvalu 84, 15006 Prague, Czech Republic

**Keywords:** Thromboelastometry, Tracheostomy, Plasma, Bleeding, Sepsis

## Abstract

**Background:**

Coagulopathy is often accompanied by prolongation of prothrombin time (PT) in septic and nonseptic patients in intensive care unit (ICU). The conventional way to correct the coagulopathy is to administer fresh frozen plasma (FFP) before invasive procedures to minimise the risk of bleeding. However, prolonged PT can be present even in hypercoagulation status, resulting in unnecessary administration of FFP. In the present study, we have assessed the reliability of thromboelastometry in case of prolonged PT and the relationship to bleeding complications during surgical tracheostomy.

**Methods:**

The study was conducted during the period between April 2013 and April 2014 in patients undergoing surgical tracheostomy. Coagulation status was assessed using PT, and the status was reassessed by thromboelastometry for prolonged PT. Tracheostomy was performed in patients with normal thromboelastometry results without administering FFP.

**Results:**

Tracheostomy was performed in total 119 patients. Normal value of PT as measured by international normalized ratio (INR) ≤ 1.2 was found in 64 (54 %) patients, while prolonged INR > 1.2 was found in 55 (46 %) patients. Patients with INR ≥ 1.3 (with INR min- 1.3, max- 1.84, and median- 1.48) were further analysed by thromboelastometry. Despite prolonged INR, thromboelastometry results were in normal ranges in all cases except one. With normal thromboelastometry, tracheostomy was performed safely without any bleeding complication.

**Conclusions:**

Surgical tracheostomy in septic and nonseptic patients can be performed without bleeding complications in case of normal thromboelastometry results (EXTEM CT) despite increased PT-INR. This method can help physicians to reduce unnecessary administration of FFP in patients.

## Background

Coagulopathy is a well-known phenomenon among intensive care unit (ICU) patients undergoing surgical procedures such as tracheostomy [1]. It is often accompanied by prolonged standard coagulation tests such as prothrombin time (PT, international normalized ratio, INR). However, prolonged PT-INR does not necessarily indicate hypocoagulation status; it might even accompany hypercoagulation status [2]. According to the recent guidelines of the European Society of Anaesthesiology (ESA) for the management of perioperative bleeding [3], correcting prolonged PT-INR up to 1.5 is not recommended. However, in practice, even if the PT-INR is below or over 1.5, many physicians try to correct this coagulopathy, usually by administering fresh frozen plasma (FFP) prior to the planned surgical intervention including tracheostomy to reduce the risk of bleeding. Indeed, the consequences of eventual bleeding can be life threatening, even when PT-INR is below 1.5. The limitation of the PT-INR test is its inability to determine the role of other blood components, such as platelets and erythrocytes, which play important roles in the initiation of blood coagulation. Moreover, the PT-INR does not provide any information on other coagulation phases, such as propagation, clot strength and fibrinolysis, which can be decreased or increased despite of normal or pathologic PT-INR of 1.5. Therefore, surgery always remains associated with the bleeding risk irrespective of patients’ PT-INR status. Rotational thromboelastometry (ROTEM) as viscoelastic method may be a better method in this regard and can be used for differentiating hypocoagulation from hypercoagulation situations [2] thereby assessing coagulation status better than the PT-INR. Thus, ROTEM could help in providing aimed administration of FFP and other drugs to patients who are at risk of bleeding even before surgical complication. Furthermore, with such a reliable method of coagulation monitoring, it would be possible to reduce transfusion of FFP and minimise risks associated with FFP administration [4]. In this study, our objective was to find out whether surgical tracheostomy can be done safely and without bleeding complication in patients with normal ROTEM-EXTEM results despite prolonged PT-INR in ICU patients.

## Methods

This prospective study was approved by the Ethics Committee for Multi-Centric Clinical Trials of the University Hospital Motol. In all unconscious patients informed consent was obtained from family members. One hundred nineteen patients, who underwent planned surgical tracheostomy at the Department of Anaesthesiology and Intensive Care Medicine, Motol University Hospital in Prague between April 2013 and April 2014, were enrolled for this study. Blood coagulation status was assessed by PT–INR, analysed with CA-7000 automated coagulation analyser (Sysmex Co., Kobe, Japan). Arterial blood was used for this analysis. In the cases where PT-INR was prolonged over 1.2, while the platelets count was higher than 50 × 10^9^/L and activated partial thromboplastin time was normal (APTT-INR 0.8-1.18), ROTEM-EXTEM (TEM International GmbH, Munich, Germany) was also performed. Tracheostomy without prior administering FFP was performed in patients with at least minimal values of normal range for EXTEM results (coagulation time - CT, clot formation time - CFT, alpha-angle, A10 – amplitude in mm 10 min after CT, maximum clot firmness - MCF, lysis index - LI30 and LI60). In order to correct coagulopathy before tracheostomy, 4 FFP was administered only in those cases where ROTEM revealed pathological values in EXTEM CT. ROTEM was not re-assessed after the intervention. Bleeding was assessed during tracheostomy as well as 24 h after the procedure. The following three conditions were considered as complication: (i) surgeon performing tracheostomy assessed bleeding as major or nonstandard, (ii) surgical revision was necessary to stop bleeding, or (iii) decrease in haemoglobin levels during 24-hour postoperative period because of tracheal bleeding. We also identified the cause for which the patients were brought to ICU and accordingly**,** divided them into two groups as septic (patients who were admitted to ICU because of severe sepsis) and nonseptic (admitted for a reason other than sepsis). Coagulation status was compared between the two study groups not only to find out whether there was any difference in ROTEM-EXTEM (tissue factor-triggered extrinsic pathway) findings between two groups, but also to check whether prolonged PT- INR was connected to sepsis in the participants.

### Statistical analysis

For data analysis, we used GraphPad Prism 6.0 statistics program. While descriptive statistics was used to determine minimal, maximal and median values, unpaired two tailed *t*-test was used to compare EXTEM parameters and coagulation parameters between PT-INR 1.21-1.29 and PT-INR >1.3 groups, and between nonsepsis and sepsis groups (*p* < 0.05 was considered as significant).

## Results

**Fig. 1:**
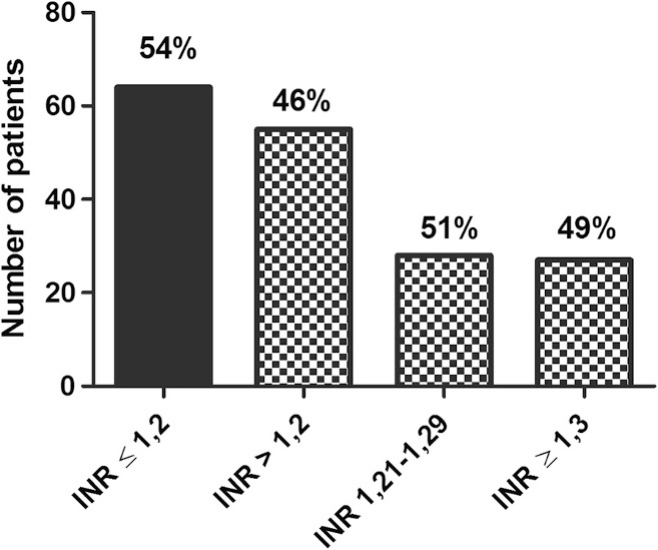
Frequency of patients with laboratory coagulopathy represented by INR (INR - International normalized ratio)

During the study period (April 2013–April 2014), otorhinolaryngologist surgeons performed 119 tracheostomies in those ICU patients who needed artificial mechanical ventilation even after tenth day of their hospitalisation. Normal value of PT-INR ≤ 1.2 was found in 64 (54 %) patients, while prolonged PT-INR over 1.2 was found in 55 (46 %) patients (Fig. 1). ROTEM-EXTEM was performed only when PT-INR was ≥ 1.3 (PT-INR min 1.3, max 1.84, and median 1.48), because neither the surgeon nor the physician would correct PT-INR below 1.3. We found 28 (51 %) patients with PT-INR value between 1.21–1.29 and 27 (49 %) patients with PT-INR ≥ 1.3 (Fig. 1). Except in one patient (EXTEM CT of 99 s, PT-INR 1.4, APTT-INR 1.18), EXTEM results were in normal ranges or hypercoagulable in almost all patients with PT-INR ≥ 1.3 (Table 1). Tracheostomy was performed safely without any bleeding complication in patients with normal EXTEM results.

**Table 1:** ROTEM-EXTEM and laboratory coagulation parameters in examined patients

EXTEM parameters	INR 1.21-1.29 (mean ± SD)	INR ≥ 1.3 (mean ± SD)	Nonsepsis (mean ± SD)	Sepsis (mean ± SD)
CT (seconds)	58.60 ± 14.01	60.79 ± 7.58 ^ns^	58.75 ± 10.42	61.92 ± 7.29 ^ns^
CFT (seconds)	50.40 ± 21.41	67.05 ± 24.98 ^ns^	71.17 ± 27.75	56.00 ± 19.8 ^ns^
α angle (degrees)	80.60 ± 4.04	76.79 ± 4.67 ^ns^	76.50 ± 5.27	78.67 ± 4.8 ^ns^
A10 (mm)	69.00 ± 9.67	65.16 ± 8.02 ^ns^	62.75 ± 8.98	69.17 ± 6.44 ^p=0.056^
MCF (mm)	73.2 ± 8.17	70.47 ± 6.02 ^ns^	68.33 ± 6.91	73.75 ± 4.75 *
LI30 (%)	99.60 ± 0.55	99.63 ± 0.59 ^ns^	99.58 ± 0.67	99.67 ± 0.49 ^ns^
LI60 (%)	94.00 ± 4.12	96.47 ± 3.47 ^ns^	93.50 ± 3.56	98.42 ± 1.51 ***
ML (%)	9.00 ± 5.61	6.21 ± 4.93 ^ns^	9.67 ± 4.89	3.92 ± 3.48 **
Laboratory parameters				
Fibrinogen g/L	4.57 ± 0.88	4.73 ± 6.62 ^ns^	4.72 ± 0.53	4.68 ± 0.79 ^ns^
Platelets(x10^9^/L)	209.4 ± 132.5	160.1 ± 77.18 ^ns^	164.8 ± 101.1	175.9 ± 81.99 ^ns^

CT – coagulation time, time from the start of the sample run to the first detectable clot formation (amplitude =2 mm); CFT – clot formation time, time from CT to the clot amplitude of 20 mm (to specify the kinetics of the clot development); α angle, angle between the trace and the x-axis; A10 clot firmness (in mm amplitude) at the respective, time point after CT

MCF- maximum clot firmness; LI30 and LI60 - Lysis Index at time 30 and 60 min after CT; ML - maximum lysis during whole measuring time of (90 min)

Normal values for ROTEM: EXTEM – CT 38–79 s, CFT 34–159 s, α angle 63-83°, MCF 50–72 mm, LI30 0-7 %, LI60 0-15 %; Fibrinogen- 1.8-4.2 g/L, Platelets count – 150-400x10^9^/L

ns - means nonsignificant difference between two groups *p* > 0.05 * means significant difference between two groups *p* < 0.05 ** means significant difference between two groups *p* < 0.01 *** means significant difference between 2 groups *p* < 0.001

**Fig. 2:**
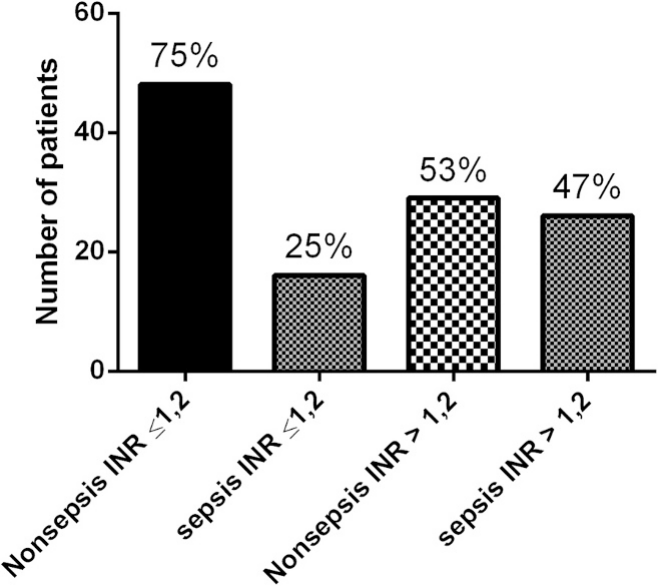
Frequency of INR in nonseptic and septic group of patients

**Fig. 3:**
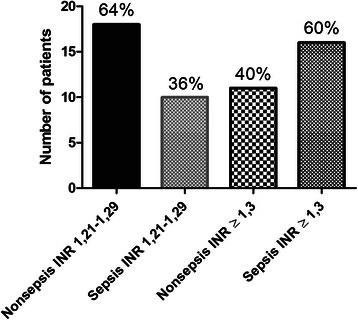
Frequency pathologic INR in nonseptic and septic group of patients

**Fig. 4:**
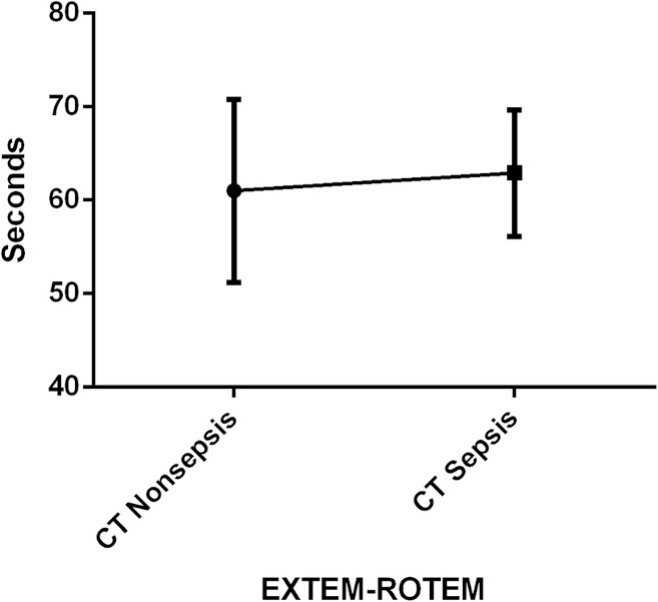
Coagulation time (CT) ROTEM parameter in nonsepsis and sepsis group of patients (INR ≥ 1.3). There is no statistical difference between CT of nonseptic and septic patients; *p* = 0.6

While analysing these ICU patients for the cause of hospitalisation, we found that sepsis counted for only 25 % patients in the group with PT-INR ≤ 1.2; nonseptic condition was the cause of hospitalisation in the rest 75 % patients in that group (Fig. 2). However, in the group with PT-INR > 1.2, 47 % patients were hospitalised for sepsis, while 53 % for nonseptic conditions (Fig. 2). In the subgroup with PT-INR 1.21–1.29, 36 % patients were with sepsis, while 64 % were with nonseptic conditions (Fig. 3). Interestingly, in the subgroup with PT-INR ≥ 1.3, nonseptic condition was present in only 40 %, patients with sepsis increased to 60 % (Fig. 3). However, we did not find any statistical difference between the septic and nonseptic initiation phase of coagulation (p =0.6) as represented by CT of ROTEM-EXTEM in the group with PT-INR ≥ 1.3 (Fig. 4). As well, comparing overall septic group to nonseptic group there was no significant difference in parameters of CFT, alpha angle (*p* > 0.05), but when looking closer to cloth strength, parameter A10 was borderline (p = 0.056), MCF was significantly higher in septic patients (p < 0.05) and fibrinolysis was significantly decreased in sepsis (p < 0.05) represented by parameters LI60 and ML.

## Discussion

Coagulopathy in ICU patients is often reported in literature, but there is an ongoing debate about its pathophysiology. Kinasewitz et al. described that the consumption and depletion of endogenous haemostasis factors in sepsis result in the prolongation of PT-INR [5]. On the other hand, platelets are often activated by inflammatory process [6] together with increased level of fibrinogen [7]; this situation can often lead to hypercoagulation and fibrin deposition connected to organ dysfunction [8–10]. However, in inflammation and sepsis hypercoagulation can quickly change to hypocoagulation, while PT-INR itself remains unchanged. As PT-INR does not take into account all coagulation factors, thromboelastography/thromboelastometry (TEG/ROTEM) seems to be a better method for differentiating between those two stages (hyper vs. hypocoagulation) in septic and nonseptic patients [2, 11–13].

Our results of EXTEM say that if parameters such as CT, CFT, alpha angle, A10, MCF, LI30, LI60, ML are in normal range or hypercoagulable, surgical procedure such as tracheostomy can be done safely. These findings are similar to recently published data of Greene et al. demonstrating that thromboelastometry measures of clot firmness (EXTEM A10, A20, and MCF) are superior to platelet count in predicting bleeding in patients with severe thrombocytopenia [14]. Our finding of decreased later fibrinolysis in septic patients represented by LI60 and ML is in accordance with similar results of Amazik at al. [15].

To the best of our knowledge, the present study is the first report where ROTEM-EXTEM was used as a global coagulation test for evaluating of coagulation status before surgical tracheostomy and comparing to standard coagulation tests. Here we provided mostly the descriptive statistics (Figs. 1, 2 and 3), because it would be unethical to look for a point in ROTEM at which the patient would start bleeding as bleeding is a life-threatening risk. Data obtained from descriptive analysis are sufficient to provide both clinical and forensic supports to the surgeon as well as to the physician taking care of the patient and that can lead to decreased administration of unnecessary FFP administration.

When EXTEM CT was pathologic, 4 units FFP were administered to the patient before tracheostomy. As we did not do control ROTEM, we do not know if FFP was able to correct prolonged CT. However, many authors say that FFP is not able to correct a mild increased PT-INR of 1.3 to 1.8 [16–18]. There was only one case when EXTEM CT was prolonged (CT of 99 s, PT-INR was 1.4 and APTT-INR was 1.18) out of 27 ROTEM tests performed in the subgroup of PT-INR ≥ 1.3.

Figure 1 shows that among the 119 patients who underwent tracheostomy, normal PT-INR (≤1.2) was present in 54 % of cases and pathologic PT-INR (>1.2) in 46 % of cases. The group with pathologic PT-INR was further divided into two subgroups with PT-INR 1.21–1.29 and PT-INR ≥ 1.3. This is because, in practice, a physician would not correct PT-INR up to 1.29; hence, we performed EXTEM only for the subgroup with PT-INR ≥ 1.3. Interestingly, the number of patients was almost identical in both subgroups, i.e., 51 % of patients with PT-INR over 1.2 belonged to the group with PT-INR 1.21–1.29 and 49 % belonged to the other group (Fig. 1).

Next we attempted to find whether any association exists between the prolonged PT-INR and the reasons for hospitalisation. Based on our analysis, we divided the patients into two groups: (i) septic group and (ii) nonseptic group (Table 2). Nonsepsis patients prevailed in the study group with PT-INR ≤ 1.2, while the group with PT-INR > 1.2 had 53 % nonseptic patients and 47 % septic patients (Fig. 2). Therefore, it is evident that coagulopathy with prolonged PT-INR was almost identically present in septic and nonseptic patients. Interestingly, in the subgroup of PT-INR 1.21–1.29, nonsepsis prevailed in 64 % of patients over 36 % sepsis patients. However, when PT-INR exceeded 1.29, the coagulopathy proportion turned around with 60 % patients with PT-INR ≥ 1.3 found in the septic group; the residual 40 % were in nonseptic group (Fig. 3).

**Table 2:** Causes of nonsepsis and sepsis which brought patients to ICU

	Causes	No. of patients brought to ICU
Nonsepsis	Polytrauma	24
	Cranial trauma	10
	Hemorrhagic stroke	14
	Ischemic stroke	4
	Cardiopulmonary resuscitation	13
	Brain metastasis	3
	Lung transplantation	2
	Cervical spine Injury	3
	Ileus without sepsis	2
	Poisoning:	
	Carbon monoxide	1
	Ethylene glycol	1
Sepsis	Pneumonia	24
	Peritonitis	10
	Coxitis	1
	Mediastinitis	1
	Gonitis	1
	Pyelonephritis	1
	Pancreatitis	1
	Spondylodiscitis	1
	Cholecystitis	1
	Gangrene of lower extremity	1

The normalization of EXTEM CT despite an increased PT-INR value might be due to the presence of sepsis with increased tissue factor expression on circulating monocytes [15, 19, 20] and this cannot be detected in plasmatic coagulation tests such as PT-INR since the cells are removed by centrifugation, however, CT in ROTEM can be shortened in this case. Coagulation time of EXTEM in PT-INR ≥ 1.3 subgroup did not differ statistically (*p* > 0.05) between those two groups; it was, in fact, in the normal range. No patients had bleeding complication in either group despite the prolonged PT-INR, which represent mainly clotting factor deficiency. This could be explained by the fact that despite the coagulation factor deficiency in ICU patients, other substances of blood important in coagulation such as platelets, fibrinogen, etc., might compensate for factor insufficiency.

Beiderlinden at al. found that bleeding incidence during and following dilatational tracheostomy was higher in patients with prolonged APTT and with platelets below 50 × 10^9^/L [21]. This may be true as platelets below 50 × 10^9^/L are generally connected with prolonged bleeding time and impaired primary hemostasis. That is why our patients had at least this number of platelets. Notably, platelet dysfunction due to antiplatelet drugs cannot be detected by ROTEM. However, Greene et al. demonstrated that ROTEM clot firmness (A10 or MCF in INTEM or EXTEM) is superior to platelet count in predicting bleeding in pediatric patients

with ITP (Immune Thrombocytopenia) and severe thrombocytopenia (<30x10^9^/L) [14]. Furthermore, Fayed et al. reported that a ROTEM-guided transfusion protocol in patients undergoing liver transplantation can avoid 75 % of platelet transfusions in patients with a platelet count < 50 × 10^9^/L without increased bleeding rate [22]. Therefore, clot firmness (A10 or MCF) should be considered in addition to CT in particular in patients with thrombocytopenia. When it comes to APTT, we excluded patients with prolonged APTT for the following reasons: (i), extrinsic pathway of coagulation initiation prevails in vivo, as described in cellular model of coagulation; (ii) in our chronic ICU patients PT prolongation is more common than APTT; (iii) when PT is prolonged, prothrombin complex can be used instead of FFP. Prolonged APTT is a more complex concern as it can be caused by heparin or hemophilia but we plan to look closer to this problem in the future.

The limitation of the study is the fact that at the time of performed tracheostomy the primary cause and reason for admission to ICU had almost resolved (tracheostomy is usually performed after tenth day of artificial ventilation) and patients from both groups were becoming alike, meaning becoming patients with ICU coagulopathy. That might be the reason why there is little and no statistically significant difference in CT-EXTEM parameters between groups. Another limitation of the study is the number of subjects involved in the study but as a pilot study it can be useful for other researchers also in other medical fields.

## Conclusions

Based on the above findings, we conclude that thromboelastometry (ROTEM-EXTEM) is a reliable method for assessing coagulation status in patients in ICU and it is superior to standard coagulation prothrombin time (PT-INR). Thromboelastometry can differentiate hypocoagulation status from normal/hypercoagulation status and thus even in case of prolonged PT-INR surgical tracheostomy can be performed safely without bleeding complication in septic and nonseptic patients provided that EXTEM parameters are at least normal. This method also supports physicians in reducing unnecessary FFP administration used preventively to minimise bleeding risk in patients with prolonged PT-INR.

## Abbreviations

CT: Coagulation time (time from starting the test until a clot firmness of 2 mm is reached)
ESA: European Society of Anaesthesiology
EXTEM: Thromboelastometric test with extrinsic activation by tissue factor
FFP: Fresh frozen plasma
ICU: Intensive care unit
INR: International normalised ratio
PT: Prothrombin time
ROTEM: Rotational thromboelastometry
TEG: Thromboelastography
